# Carbon Dots Conjugated Antibody as an Effective FRET-Based Biosensor for Progesterone Hormone Screening

**DOI:** 10.3390/bios12110993

**Published:** 2022-11-09

**Authors:** Disha, Poonam Kumari, Manoj K. Patel, Parveen Kumar, Manoj K. Nayak

**Affiliations:** 1 Materials Science and Sensor Applications, CSIR-Central Scientific Instruments Organisation (CSIR-CSIO), Sector 30-C, Chandigarh 160030, India; 2 Academy of Scientific and Innovative Research (AcSIR), Ghaziabad 201002, India; 3 Manufacturing Science and lnstrumentation, CSIR-Central Scientific Instruments Organisation (CSIR-CSIO), Sector 30-C, Chandigarh 160030, India; 4 Exigo Recycling Pvt. Ltd., Noida 201309, India

**Keywords:** endocrine, hormonal imbalance, progesterone, biorecognition, immunosensor, fluorescence resonance energy transfer (FRET) bioassay

## Abstract

In this work, carbon dots (CDs) were synthesized by a one-step hydrothermal method using citric acid and ethylene diamine, and covalently functionalized with antibodies for the sensing of progesterone hormone. The structural and morphological analysis reveals that the synthesized CDs are of average size (diameter 8–10 nm) and the surface functionalities are confirmed by XPS, XRD and FT-IR. Further graphene oxide (GO) is used as a quencher due to the fluorescence resonance energy transfer (FRET) mechanism, whereas the presence of the analyte progesterone turns on the fluorescence because of displacement of GO from the surface of CDs effectively inhibiting FRET efficiency due to the increased distance between donor and acceptor moieties. The linear curve is obtained with different progesterone concentrations with 13.8 nM detection limits (R^2^ = 0.974). The proposed optical method demonstrated high selectivity performance in the presence of structurally resembling interfering compounds. The PL intensity increased linearly with the increased progesterone concentration range (10–900 nM) under the optimal experimental parameters. The developed level-free immunosensor has emerged as a potential platform for simplified progesterone analysis due to the high selectivity performance and good recovery in different samples of spiked water.

## 1. Introduction

A biologically active hormone called progesterone occurs as a natural estrogenic steroid compound, derived from a cholesterol biosynthesis [1]. This multifunctional hormone is associated with the reproductive aspects and sexual growth, the maintenance of the menstrual cycle and pregnancy. Additionally, progesterone is having crucial pharmaceutical applications where it is implemented as oral contraceptives, menopausal hormone therapy, infertility, etc. [2]. Progesterone has been reported to be a significant carcinogen, its abnormally high levels can be toxic to both men and women and as tumorgenic, affecting human health by interfering with the natural hormonal activity due to excessive exposure during gestation. Indeed, steroid estrogens have gained significant attention in recent years due to their rapid rise in levels posing serious threats to soil, plants, water resources and humans [3]. Over the years, the analysis of progesterone in environmental samples has been considerably dominant because of its endocrine disrupting activity [1,4,5]. Accordingly, the detection of progesterone hormone is becoming essential for environmental analysis.

In recent years, several methods have influenced the analysis of progesterone by using chromatographic techniques like gas chromatography (GC) [1] and liquid chromatography (LC) coupled with mass spectrometry (LC-MS) [6] and high performance liquid chromatography (HPLC) [7]. However, these techniques require repeated measurements, long and cumbersome derivatization processes for sample preparation, and trained operators, leading to their inappropriate field applications. Additionally, the enzyme linked immunosorbent assay (ELISA), radioimmune assays, etc., come with their lingering challenges of exorbitant instrumentation prices and limited stability [8]. At present, the electrochemical techniques have also been emerged as a potential approach where steroid hormones are being quantified even at low levels of concentration with high sensitivity [2,9,10]. Additionally, molecularly imprinted polymers (MIPs)-based approaches have produced many opportunities for the detection, quantification and removal of estrogenic steroid hormones [11,12,13]. However, the molecular imprinting technology remained challenging and needs to be simplified in their synthesis, maintaining shape and porosity before and after template removal and binding procedures [1,14]. In this context, Table 1 represents the literature where CDs being exploited in progesterone determination in these past years are yet to be explored in an interference analysis or specificity performance with their analogous hormones or biomolecules such as β-estradiol, testosterone, cortisol, bisphenol A, etc. for applications in complex matrices [15,16,17,18]. 

Simultaneously, the fluorescence spectroscopic methods provided unique advantages for bioanalysis applications owing to the simplicity of their operation, stability and sensitivity [19,20]. A renowned technology called fluorescence resonance energy transfer (FRET) garnered a considerable interest for the sensing analysis of biological molecules. FRET is described as a phenomenon in which the energy transfer occurs non-radiatively from the excited fluorophore donor to the acceptor of the ground state by dipole-dipole coupling [21,22]. The efficiency and sensitivity of the FRET are significantly enhanced by the combination of intermolecular dipole–dipole interactions between donor planarized geometries and acceptors in the resulting FRET process. In particular, chemically stable aqueous carbon dots (CDs) have superior advantages over the fluorescent organic dyes and other semiconductor quantum dots. Likewise, Junkai Ren et al. have presented the structural design and optimization of CD-based nanocomposites to meet the custom application aimed at avoiding quenching effects and improving the range of optical solid-state responses. Having the precise control over CDs’ structural and chemical characteristics, solid-state can develop high performance devices by competing semiconductors and quantum dots [23]. Since CDs apparently have tunable fluorescence, nontoxicity, remarkable photostability, biocompatibility and ease to be modified with biomolecules, are therefore used worldwide for fluorescent detection strategies [24,25].

In contrast with the conventional CDs and other semiconductors, the fascinating physicochemical properties of nitrogen-doped fluorescent CDs such as biocompatibility and water solubility have opened a new horizon in the bio sensing era based on FRET applications. Moreover, the presence of abundant amine and carboxylic acid groups at the surface of nanoparticles provides a number of binding regions for conjugating antibodies. The emerging applications of nitrogen-doped CDs in FRET-based biosensors impart enhanced quenching efficiency to the system which is advantageous for developing a potential fluorescent technique [26]. The use of nanoparticles' distinctive optical quenching characteristics such as gold nanoparticles (Au NPs), transition metal dichalcogenides (TMDCs), graphene oxide (GO), etc., as an effective fluorescence quencher in FRET-based biosensors has been illustrated in the literature [22,26,27]. However, GO exhibits an outstanding quenching ability as an acceptor, owing to its wide electronic bands of absorption as well as the sp^2^ hybridized monolayer structure [28]. Optical immunoassays using fluorescence have reached great interest in nanotechnology for the development of biosensors because of their sensitivity and selectivity. The literature has also presented number of reports for monitoring steroid hormones using FRET as a transduction part for biosensing approach [29,30]. However, the detection of FRET-based progesterone hormone using a donor-acceptor combination of CD and GO is in the early stages of investigation. Recently, a significant and sensitive FRET aptasensor for progesterone based on carbon dots has been reported by H. Cui et al. with a reduced detection limit 3.3 × 10^−11^ M [31]. However, the detection range is limited to 120 nM in milk, which is not suitable for aqueous samples where progesterone is also present at higher levels, i.e., >120 nM [32].

Herein, we presented the use of an optical platform in order to develop a FRET-based, label-free, and selective progesterone immunoassay through the photoluminescence system. In this context, the CDs were prepared from the precursors, citric acid and ethylenediamine using a one-step hydrothermal process. Consequently, graphene oxide was synthesized from our preceding report [2]. We use a simple one-step process to get antibody-conjugated CDs for assessment of the progesterone hormone. Moreover, CDs, GO and antibody-conjugated CDs were successfully characterised with XRD, FT-IR techniques. The as-synthesized amine doped CDs were highly fluorescent and could be implemented directly to immobilize end-on oriented antibodies. Further, we reported an optical technique in which π-π interactions between CDs and GO effectively quenched the fluorescence of CDs and antibody-conjugated CDs (Ab-CDs) in the absence of the target analyte i.e., progesterone. However, in the presence of progesterone molecules, the latter bound specifically to the corresponding antibodies on the CDs. As a result, increasing the distance between the quencher and the donor pair of the FRET process, recovers the fluorescent CDs. The analysis of the developed progesterone fluorescent assay based on GO and CDs demonstrated a remarkable selectivity in the presence of several analogous biomolecules. The current FRET-based biosensor for progesterone detection has demonstrated a straightforward and cost-effective methodology with high selectivity in complex aquatic samples. Moreover, the approach is devoid of keeping long incubations and provides opportunities in various other bioanalytical applications. 

## 2. Experimental Sections

### 2.1. Materials and Solutions

Citric acid, Ethylenediamine, 1-ethyl-3-(3dimethylamino-propyl) carbodiimide (EDC), *N*-hydroxysuccinimide (NHS), Bisphenol A, β-estradiol, Testosterone, Cortisol, Progesterone hormone and its monoclonal antibody and other reagents were obtained from Sigma-Aldrich, Bangalore, India. All experimental solutions including phosphate buffered saline (PBS, 10 mM, pH 7.4) were carefully made with double distilled water (DDI).

### 2.2. Instrumentation

A Nicolet iS10 (Thermo-Scientific, Portland, OR, USA), Fourier Transform Infrared (FT-IR) was used to obtain an infrared absorption spectrum of solid samples. A Jeol/JEM 2100 (Tokyo, Japan), high-resolution transmission electron microscope (HR-TEM) and X-ray diffractometer (XRD) of Bruker AXS, Karlsruhe, Germany, D8 Advance (Cu Kα λ = 1.5406 Å) were used to obtain the morphology of carbonaceous nanomaterials and XRD pattern respectively. X-ray photoelectron spectra (XPS) was observed using a Thermo-Scientific (Portland, OR, USA) K-Alpha XPS for the chemical structure and composition of CDs. The fluorescence and whole of the optical experiments of CDs were recorded using a Cary Eclipse fluorescence spectrophotometer (AGILENT, Santa Clara, CA, USA) equipped with a xenon lamp using right angle geometry. The excitation and emission slit widths were set at 4 nm each. The fluorescence measurements covered excitation wavelengths from 320 to 380 nm and emission wavelengths from 10 nm above each excitation wavelength to 600 nm in 1 nm increments. The absorption spectra of CDs and GO were obtained using (PerkinElmer Lamda 850, Shelton, CT, USA) UV-vis spectrophotometer.

### 2.3. Synthesis of Carbon Dots

Nitrogen-doped CDs were synthesized using a one-step hydrothermal method using citric acid as a carbon precursor and ethylenediamine as a nitrogen dopant [19]. Briefly, 2 g of citric acid and 2.55 mL of ethylenediamine were dispersed in 20 mL of DI water and then transferred to the Teflon bottle in order to heat the reaction mixture at 200 °C for 5 h. The obtained pale-yellow solution was naturally cooled and filtered through a 0.22 μm filter membrane and dialysed for 24 h with a 0.5 K molecular weight cut-off (MWCO) dialysis bag by changing water at intervals of 12 h to obtain a solution of carbon dots. The nitrogen-doped CD solution was diluted and stored at 4 °C before further experimental use. GO were prepared using the modified Hummer method as described earlier [2].

#### Synthesis of Antibody Conjugated Carbon Dots (Ab-CDs)

CDs–antibody conjugated nanomaterials were prepared according to a reported method with some modifications [33]. The EDC–NHS-induced bioconjugation reaction was used to covalently bind antibodies to the functional groups of CDs. The 0.125 mL antibody solution (50 µg/mL) was incubated with 0.2 mL 100 mM (1:1) molar ratio of EDC–NHS solution at pH 7.4 for ½ hour to activate the COOH groups on the antibodies. The activated antibodies were then incubated with 0.4 mL of CDs (2 mg/mL) for 2 h at 37 °C to form CD–antibody complexes. In this process, CDs and progesterone antibodies were conjugated through strong amide bonds between the amine groups of CD nanoparticles and the carboxylic acid groups of antibodies. The aforementioned solution was dialysed and stored at 4 °C prior to use.

## 3. Results and Discussion

### 3.1. Characterization

**Figure 1:**
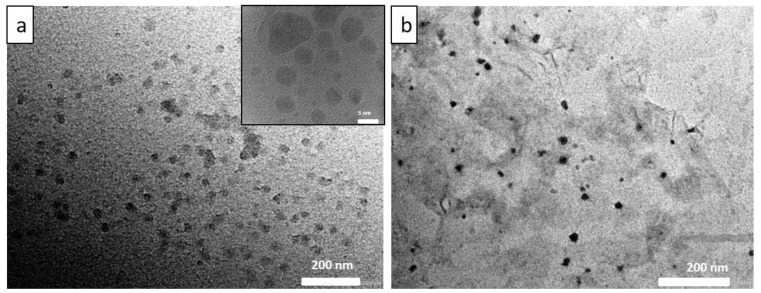
The representative HRTEM images of CDs at different scales (**a**) and CDs absorbed on GO surface (**b**).

**Figure 2:**
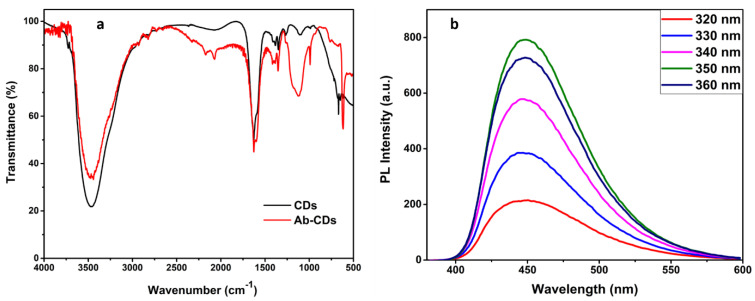
FTIR spectra of CDs and Ab−conjugated CDs (**a**) and PL graph of CDs at different excitation wavelengths (**b**).

As per our previous report, GO exhibiting excellent dispersibility was obtained from modified Hummer’s method [2]. The experimental data on GO synthesis and characterization are discussed in the supplementary information (Figures S1 and S2 in supplementary materials). CDs, synthesized by hydrothermally treating citric acid and ethylene diamine also reveal good aqueous solubility. Hence, the nanomaterials were characterized using several analytical techniques including UV, PL, FTIR, XPS, XRD, HRTEM, etc., to confirm the chemical structure and characteristics of the surface functional groups and optical behaviour. The structural morphology of the CD samples was elucidated using HR–TEM and shown in the Figure 1a, revealed that CDs are spherically shaped and have average particle sizes, ~8 nm. Furthermore, CDs absorbed on layers of GO is clearly observed in Figure 1b. In addition, the histogram for the particle size distribution of CDs’ dispersion is provided in the Figure S4. FTIR spectra of *N*-doped CDs and Ab–CDs were recorded to identify the surface functional groups (Figure 2). The FTIR spectra of CDs exhibited broad vibration bands at 3465 and 3227 cm^−1^ corresponding to the functional groups, O–H and N–H stretching, respectively. The peaks observed at 2818–2929, 2084–2364, 1627 and 1592 cm^−1^ are due to the stretching of the C–H bond, the nitrile C≡N bond, the amide C=O and the N–H bending, respectively. Besides, the peaks at 1350–1384, 1100–1266, and 617–669 cm^−1^ are assigned to C–O stretching or C–H bending, C–N stretching and C–C bending vibrations in the CDs [34,35,36,37,38].

In addition, the PL analysis of as-prepared CDs is displayed in Figure 2b at a range of 320–360 nm as excitation wavelength. Since there is no significant trend in red or blue shifting of emission peaks with the increase in excitation wavelength in PL, we can clearly state the optical property of CDs as excitation-independent [39]. Moreover, the strongest fluorescence emission, at 350 excitation wavelength was demonstrated at 450 nm. However, the decrease in PL intensity along with a slight red shift in emission peak can be observed at a higher wavelength range, i.e., 380–450 nm (Figure S3). This might be due to the different trap states of surface groups and size dispersion of CDs [40]. 

**Figure 3:**
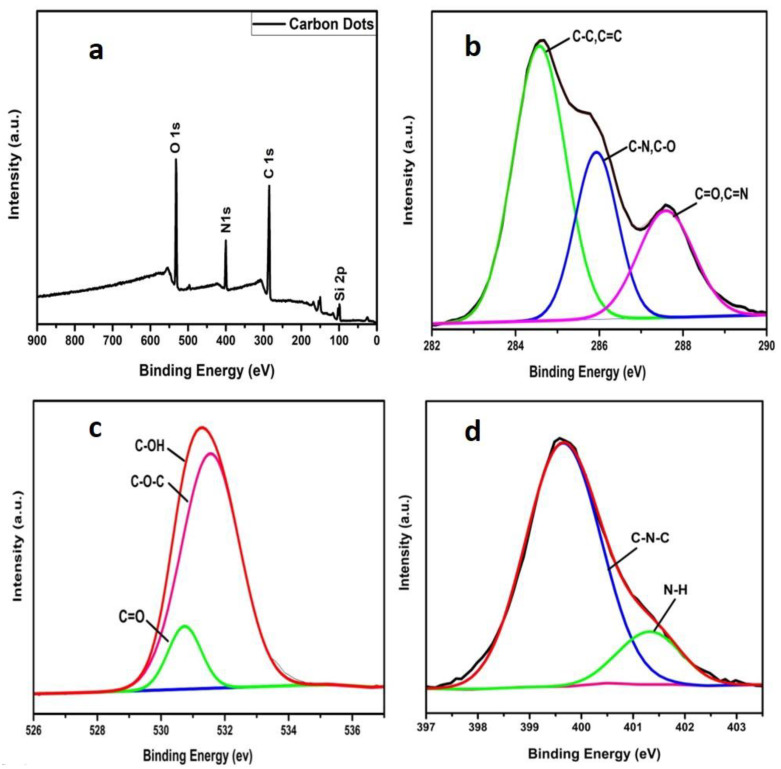
XPS graph of as prepared CDs (**a**), obtained spectra of C1s (**b**), O1s (**c**) and N1s (**d**) peaks with high resolution.

The XPS analysis was carried out for investigating the chemical as well as elemental composition of CDs which confirmed three typical peaks at 285.4 eV, 400 eV and 531.8 eV of C1s, N1s and O1s, respectively, as shown in Figure 3a. Additionally, the high resolved spectra of C1s displays a further three peaks of binding energy at 284.6, 285.9 and 287.6 eV, representing the sp^2^ graphitic structure (C=C), existing C–N/C–O and C=O/C=N respectively (Figure 3b). Similarly, characteristic peaks of O1s and N1s are presented in Figure 3c,d where N1s revealed two peaks ascribing to the graphitic carbon nitride C–N and N–H groups at the CDs’ surface. Similarly, characteristic peaks of O1s and N1s are presented in Figure 3c,d where N1s revealed two peaks ascribing to the graphitic carbon nitride C–N and N–H groups on the surface of CDs and coincides as well with FTIR results. The XPS confirms the relative % of C, O and N of CDs, i.e., 62.8%, 23.8% and 13.3%, respectively, which endows more binding sites to the nanomaterial [34,41].

**Figure 4:**
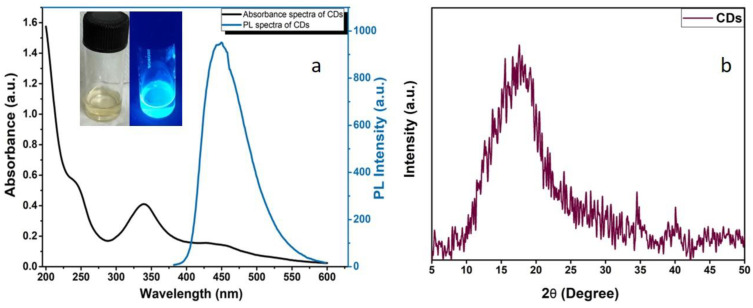
UV-PL graph of CDs and inset images of as-prepared pale-yellow coloured carbon dots solution in normal and UV light (**a**) and XRD pattern of CDs (**b**).

Figure 4a plots the absorbance as well as fluorescence spectra, where CDs exhibited a strong blue light under the ultraviolet light. The UV-Vis absorption spectrum of CDs’ aqueous suspension exhibits two absorption peaks at ~240 nm owing to the C=C bond (π-π* transition) and 340 nm of C=O bond (n-π* transitions), respectively [42]. Additionally, the absorption band expanded in the visible region at 432–450 nm is ascribed to the nitrogen-doping CDs [40]. Furthermore, the as-obtained CDs solution was pale-yellow (under visible light) and strongly emitted a blue fluorescence when kept in UV light (Figure 4 inset). The fluorescence efficiency is compared with the standard procedure currently used to determine the relative quantum yield against the standard quinine sulphate is 0.55 in 0.1 M H_2_SO_4_. The quantum yield of CDs was estimated to be 8.5% using quinine sulphate as standard. The results from XRD analysis inferred clearly that CDs have an amorphous carbon phase as there are no sharp peaks existing in the spectra but a broad diffraction peak at 2θ = 18°, Figure 4b [34,41]. 

**Figure 5:**
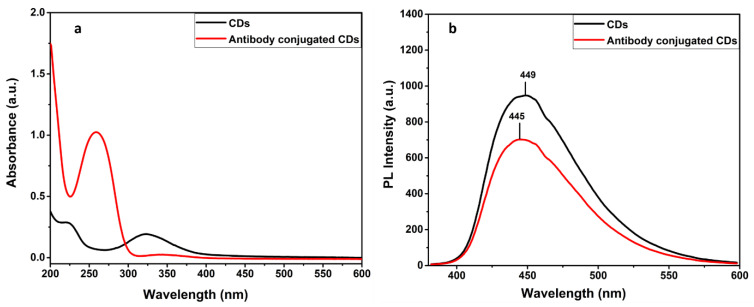
Absorbance (**a**) and PL emission spectra of CDs and antibody conjugated CDs (**b**).

The confirmation of binding the antibodies to the functional groups bearing CDs is well obtained by the UV-Vis and PL spectral analysis. Figure 5a suggests the absorption band is around 260 nm, present in CDs–antibody complexes, which is ascribed by the amino acids of the antibodies. Concentration of conjugated antibody on CDs is also estimated from the UV graph [33] (Supplementary data). Subsequently, Figure 5b indicates the decreased PL intensity with a slight blue shift ~5 nm attributing to the existence of a proteinaceous layer of conjugated antibodies. In addition, the FTIR spectra of the antibody conjugated N-doped CDs (Ab–CDs) showed broad vibration bands at 3444–3477 and 3235 cm^−1^ corresponding to the functional groups, O–H and N–H stretching respectively. The peaks observed at 2726–2928, 2071–2328, 1627 and 1601 cm^−1^ are due to the stretching of the C–H bond, the nitrile bond C≡N, the amide bond C=O and the N–H bending, respectively. Similarly, the peaks at 1353–1385, 1117–1265 and 616–764 cm^−1^ are assigned to C–O stretching or C–H bending, C–N stretching and C–C bending vibrations in the Ab–CDs. The FTIR spectra also indicate that the increase in the intensity of amide >C=O, nitride C=N, amine C–N, and aliphatic C–H bonds of the antibody conjugated Ab–CDs in comparison to CDs. The UV–Vis absorption and FTIR spectra successfully confirmed the antibody conjugation on the amine functionalized CDs of *N*-doped CDs [42,43].

#### Parametric Performance Optimization and Quenching Efficiency

**Figure 6:**
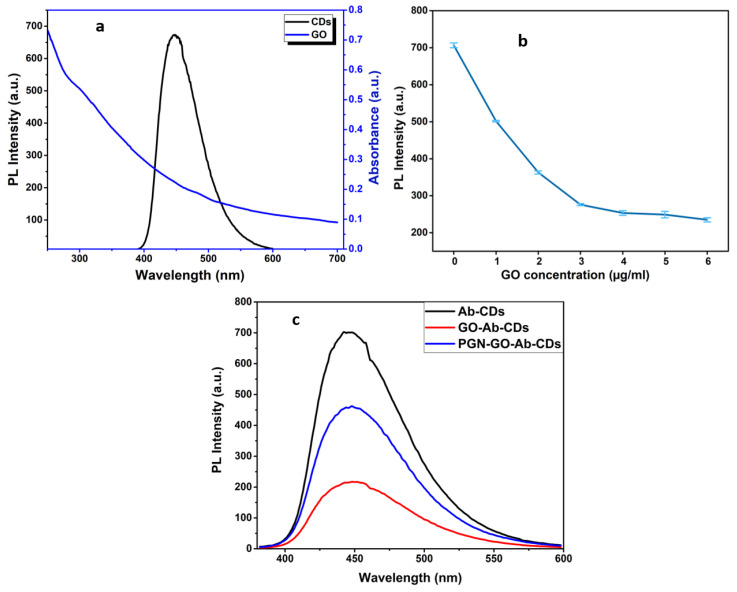
The overlapping absorbance of GO and emission of carbon dots (**a**), Investigation of the GO concentration for quenching efficiency (**b**) and Resultant PL spectra with and without the presence of quencher, GO (**c**).

As illustrated in Figure 6a, GO has a broad absorption band, allowing easy pairing and overlapping with the fluorescence emission spectra of CDs, which is advantageous for the FRET process. Consequently, GO quenched the fluorescence of the adsorbed CDs on the GO surface. Following the optimization of the GO nanosheet as Ab–CQDs emission acceptors in the FRET biosensor, several GO concentrates were examined. A total of 100 µL Ab–CDs solution was mixed with 20 µL of each GO concentrations from 1–6 µg/mL, and then recorded the fluorescence spectra for optimizing the GO amount required in effectively quenching the fluorescence of CDs. A concentration of 20 µL GO (4 µg/mL) was enough to quench the 100 µL Ab–CDs solution, so that 20 µL was selected for subsequent experiments (Figure 6b,c).

### 3.2. Development of FRET-Based Progesterone Hormone Immunosensor: Detection and Performance Analysis

An amount of 100 μL of the CDs–antibody complex solution was incubated with various concentrations of progesterone (50 μL each) from 10 nM to 900 nM for 30 min, and then this assembling solution was advanced to record PL spectra after the addition of 20 μL (4 µg/mL) GO. The total volume of 0.2 mL was composed with the PBS buffer and incubated for 5 min before recording the fluorescence spectra for batch analysis. A prerequisite for fulfilling FRET is that the donor emission spectrum must overlap with the acceptor absorption spectrum. An additional FRET prerequisite is the distance between the donor and the acceptor which should be less than 10 nm. FRET is a non-radioactive energy transfer process in which the fluorescence intensity of the donor is much lower or sometimes quenched due to the transfer of energy to the acceptor. The results show that the dominant process of fluorescence quenching is attributed to FRET occurs between CDs as a donor and GO as an acceptor which turns off strong luminescent CDs. By adding progesterone, the distance between the Ab–CDs complexes and the GO surface was increased effectively, thereby reducing the FRET effect that restored the fluorescence of the CDs.

**Figure 7:**
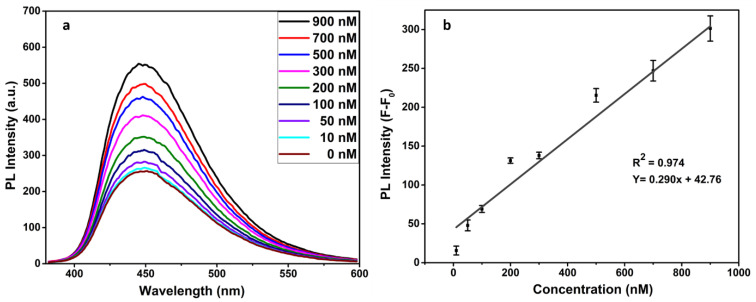
Change in PL intensity with different concentrations of progesterone hormone (**a**) and representative of a linear regression (**b**).

To demonstrate the PL response of the FRET-based immunosensor for the progesterone detection, the fluorescence spectra were recorded at an excitation wavelength of 350 nm for each variable progesterone concentration. As Figure 7 clearly shows, the PL intensity of the Ab–CD complex is somewhat attenuated by GO interactions (F_0_). While the presence of progesterone has given rise to specific bindings with antibodies that have especially increased the required distance of the FRET process between the quencher pair GO and CDs. A change in PL intensity (F-F_0_) occurred at the various concentrations of progesterone as a result of the formation of the CDs–antibody-progesterone complexes. The reason for fluorescence restoration may be that CQDs–antibody–progesterone complexes are not in immediate proximity to the GO quencher. In the absence of progesterone in solution, no characteristic change is seen in the emission peak. However, the change in PL intensity gradually increased from 10 nM to 900 nM in a linear trend (Figure 7). The detection limit of 13.8 nM was calculated using the standard deviation (S) of 3 blank readings in the formula, i.e., 3S/(SLOPE = 0.290). Consequently, the significant CDs-based FRET immunoassay was successfully employed for the selective detection of the target progesterone hormone.

#### 3.2.1. Analysing the pH Effect

**Figure 8:**
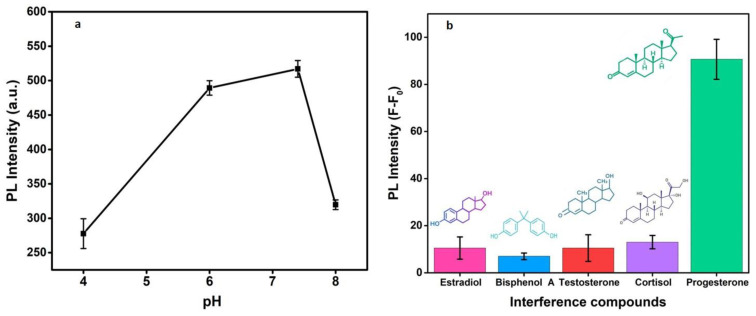
pH effect on the fluorescence of proposed PGN biosensor (**a**) and change in PL intensity with different interference compounds (**b**).

For observing the effect of pH, which is a key factor affecting the antibody bindings, buffer solutions of different pHs ranging from 4.0 to 8.0 were used for measuring the PL response. As Figure 8a infers, there is no noticeable PL response under acidic and alkaline conditions and displays maximum response at pH 7.4. This is likely a charge reversal due to the impact of pH on antibody activity and antigen uptake. Consequently, the pH of 7.4 was used as an optimized condition in this study.

#### 3.2.2. Specificity and Selectivity of the FRET Immunosensor 

Several interfering or analogous biomolecules such as β-estradiol, bisphenol A, testosterone, and cortisol were tested for the specificity as an essential parameter of the present immunosensor. As shown in Figure 8b, there is no significant change in the PL response with the potentially interfering substances at 100 nM of concentration each. However, there is a significant change in PL intensity at the same concentration of the PGN hormone. The selectivity performance of the developed optical immunoassay is ascribed to the specific antigen–antibody reactions between progesterone and its corresponding antibodies covalently conjugated on CDs. Interfering samples displayed little influence on the detection system owing to the strong immunoreactions between progesterone and Ab–CQDs, suggesting that progesterone detection could be successful in complex matrices. 

#### 3.2.3. Evaluation of FRET-Based Immunosensor Performance in Real Samples

For verifying the reliability and practicability of our fluorescence biosensor, we used the spiked water samples. The supernatant of water samples was added with progesterone of two known concentrations (100 & 200 nM) and analysed further. The 100 nM- and 200 nM-spiked samples have shown considerable rates of recovery with 95.6 (RSD = 6.5) and 98.4% (RSD = 4.5) as recovery percentages, respectively. 

## 4. Conclusions

The summary of the proposed optical methodology demonstrates the success of implementing nitrogen-doped CDs prepared from hydrothermal synthesis and GO benefits the FRET process for the detection of progesterone in aqueous samples. The prepared Ab–CDs possessed a good fluorescent quantum yield of 8.5% with quinine sulphate as a reference and an excellent photo as well as pH stabilities. The PL intensity of the CDs–antibodies conjugated system is quenched by the π-π stacking and electrostatic interactions of GO, resulting into a turn-off state. However, by adding progesterone, the strong antigenic bindings of antibodies increased the distance between the CDs–antibody complexes and GO which recovers and further modifies the PL intensity. The variation in PL intensity ranges linearly with progesterone concentrations from 10–900 nm. Based on the findings, the progesterone immunoassay indicated good analytical performance with a detection limit of 13.8 nM. Since the work is devoid of any cumbersome labelling substrate or an enzyme, it is well suited for the routine analysis of environmental water systems. Furthermore, we anticipate that this fluorescence-based rapid bio sensing approach offers various potential applications in environmental as well as complicated clinical areas. 

## Figures and Tables

**Table 1:** The summarised reported literature on sensing progesterone hormone in biological as well as environmental samples.

Sr. No.	Nanomaterials	Detection Method	Limit of Detection (LOD)	Linear Range	Detection Time	Sample	Year	Reference
1.	Pyrrole	MIP-GC	0.62 ng/mL	0.62 to 1.87 ng/mL	30 min	Blood and hospital water	2015	[1]
2.	Protein label GO-thionine	Electrochemical	6.3 × 10^−5^ ng/mL	0.02 to 20 ng/mL	100 min	Milk	2017	[9]
3.	GQDs-NiO-Au composite	Electrochemical	0.57 pg/mL (1.86 pM)	0.01 to 1000 nM	60 min	Human serum	2020	[10]
4.	CDs	Fluorescence	10.25 nM	0 to 200 μM	-	In vitro	2020	[15]
5.	CDs-GO	Photo-electrochemical	0.17 nΜ	0.5 nM to 180 nM	-	Human serum	2020	[16]
6.	QDs embedded hydrogel	Fluorescence	55 nM	78 to 632 nM	-		2020	[17]
7.	CDs	Fluorescence	9.3 nM	0 to 130 μM	1 min	Human serum	2021	[18]
8.	PdNPs/P-3TAA	MIP-electrochemical	-	0.1 nM to 110 nM	-		2022	[14]

*Abbreviations:* GC: Gas chromatography, MIP: Molecularly imprinted polymer, GQDs: Graphene quantum dots NiO: Nickel oxide, Au: Gold, PdNPs/P-3TAA: Palladium nanoparticles/Poly (thiophene-3-acetic acid).

## Supplementary Materials

The following are available online at: https://www.mdpi.com/article/10.3390/bios12110993/s1. Figure S1: HR-TEM image of GO. Figure S2: FTIR and Raman spectra of GO (a & b). Figure S3: Fluorescence spectra of CDs at higher excitation wavelengths (from 350 nm to 450 nm) (a) and corresponding normalized spectra (b). Figure S4: Histogram displaying the size distribution of CDs.
