# Supplementary material for: Carbon Dots Conjugated Antibody as an Effective FRET-Based Biosensor for Progesterone Hormone Screening
Source: Biosensors (Basel). 2022 Nov 9;12(11):993. doi: 10.3390/bios12110993 (PMC9688503; doi:10.3390/bios12110993)
Supplement: Supplementary file 1 [file biosensors-12-00993-s001.zip › biosensors-1985966-supplementary.pdf]

biosensors

MDPI

Supplementary Materials

Carbon Dots Conjugated Antibody as an Effective FRET-Based Biosensor for Progesterone Hormone Screening

Disha [1,2], Poonam Kumari [1,2], Manoj K. Patel [2,3], Parveen Kumar [4] and Manoj K. Nayak [1,2,*]

[1] Materials Science and Sensor Applications, CSIR-Central Scientific Instruments Organisation (CSIR-CSIO), Sector 30-C, Chandigarh 160030, India
[2] Academy of Scientific and Innovative Research (AcSIR), Ghaziabad 201002, India
[3] Manufacturing Science and Instrumentation, CSIR-Central Scientific Instruments Organisation (CSIR-CSIO), Sector 30-C, Chandigarh 160030, India
[4] Exigo Recycling Pvt. Ltd., Noida 201309, India
* Correspondence: mknayak@csio.res.in

Abstract: In this work, carbon dots (CDs) were synthesized by a one-step hydrothermal method using citric acid and ethylene diamine, and covalently functionalized with antibodies for the sensing of progesterone hormone. The structural and morphological analysis reveals that the synthesized CDs are of average size (diameter 8–10 nm) and the surface functionalities are confirmed by XPS, XRD and FT-IR. Further graphene oxide (GO) is used as a quencher due to the fluorescence resonance energy transfer (FRET) mechanism, whereas the presence of the analyte progesterone turns on the fluorescence because of displacement of GO from the surface of CDs effectively inhibiting FRET efficiency due to the increased distance between donor and acceptor moieties. The linear curve is obtained with different progesterone concentrations with 13.8 nM detection limits (R^2 = 0.974). The proposed optical method demonstrated high selectivity performance in the presence of structurally resembling interfering compounds. The PL intensity increased linearly with the increased progesterone concentration range (10–900 nM) under the optimal experimental parameters. The developed level-free immunosensor has emerged as a potential platform for simplified progesterone analysis due to the high selectivity performance and good recovery in different samples of spiked water.

Keywords: endocrine; hormonal imbalance; progesterone; biorecognition; immunosensor; fluorescence resonance energy transfer (FRET) bioassay

Citation: Disha; Kumari, P.; Patel, M.K.; Kumar, P.; Nayak, M.K. Carbon Dots Conjugated Antibody as an Effective FRET-Based Biosensor for Progesterone Hormone Screening. *Biosensors* **2022**, *12*, 993. https://doi.org/10.3390/bios12110993

Received: 7 October 2022
Accepted: 6 November 2022
Published: 9 November 2022

Publisher's Note: MDPI stays neutral with regard to jurisdictional claims in published maps and institutional affiliations.

Copyright: © 2022 by the authors. Submitted for possible open access publication under the terms and conditions of the Creative Commons Attribution (CC BY) license (https://creativecommons.org/licenses/by/4.0/).

1. Synthesis of Graphene Oxide (GO):

According to our previous report [1], GO was obtained following the modified Hummer's method. First, the graphite powder (500 mg) in 25 ml, H_2SO_4 was constantly stirred to mix, keeping in an ice bath. Secondly, $NaNO_3$ (500 mg) was added and continue to stir for about 2 h and subsequently $KMnO_4$ (1 g) was gently added maintaining the temperature <10 °C. The solution was stirred overnight and the next day it was refluxed at 98 °C for 24 h. After adding H_2O_2 (5 ml), the product was neutralized by thoroughly washing with an aqueous solution of 5% HCl. Finally, washed the obtained solid using ethanol and dried for 10 h in a vacuum at 60 °C.

2. Characterization:

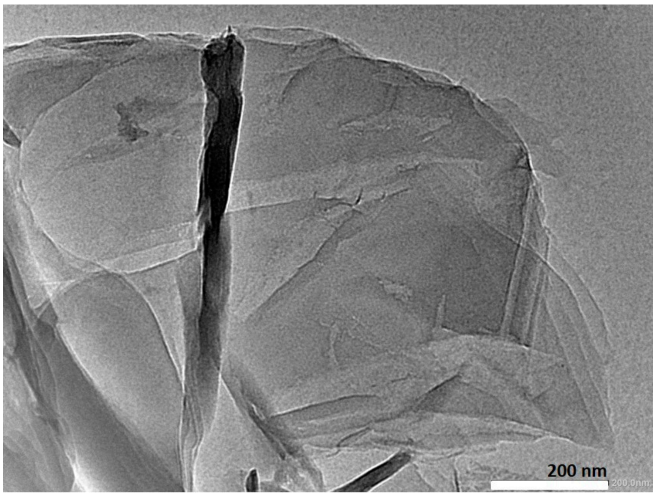

Figure S1. HR-TEM image of GO.

Figure S1 represents the HR-TEM result which inferred the sheets and layered structure of GO having stacked crumpled and folded edges. FTIR spectra showed the presence of functional groups like O-H, HOC=O, C-O, & C–O–C on GO. This is due to the occurrence of absorption peaks at 3425 cm^{-1}, 1720 cm^{-1}, 1377 cm^{-1} & 1059 cm^{-1} respectively, which also confirmed the formation of GO (Figure S2a). Additionally, Raman graph demonstrated the increased intensity of D band as more defects occurred in the carbon rings, when GO is formed (Figure S2b).

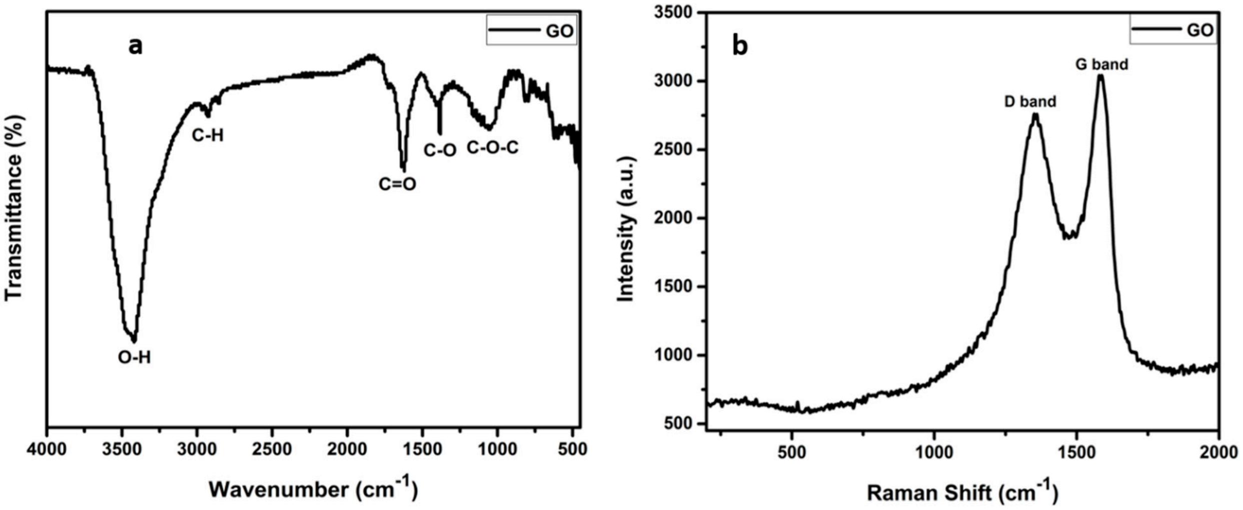

Figure S2. FTIR and Raman spectra of GO (a & b).

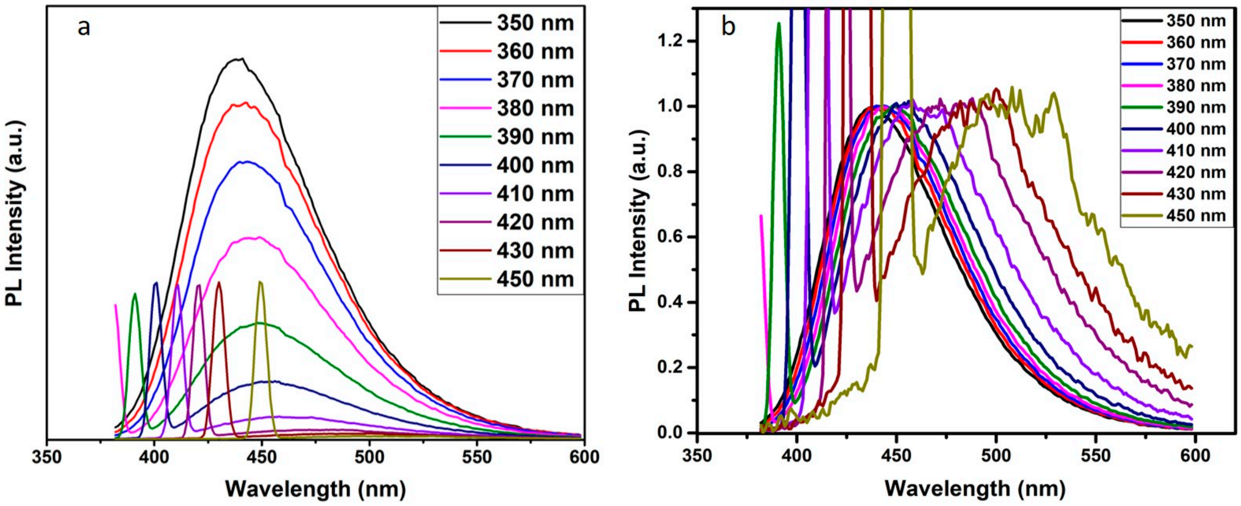

Figure S3. Fluorescence spectra of CDs at higher excitation wavelengths (from 350 nm to 450 nm) (a) and corresponding normalized spectra (b).

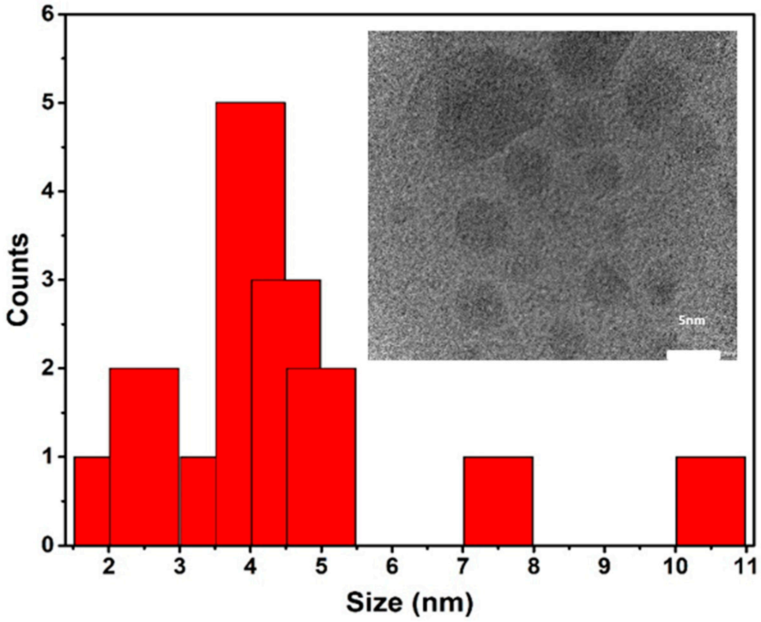

Figure S4. Histogram displaying the size distribution of CDs.

3. Calculation of antibody (protein) concentration [2].

$$\textbf{Antibody concentration \%} = A_{280} - A_{max} / A_{280} \times 100$$

Where A_{280} and A_{max} are absorbance values of antibody conjugated CDs (0.613) and CDs (0.108) respectively at 280 nm. Using the formula, the estimated protein concentration on CDs is 82.38%.

References:

1. Disha, Kumari, P.; Nayak, M. K.; Kumar, P., An electrochemical biosensing platform for progesterone hormone detection using magnetic graphene oxide. *Journal of Materials Chemistry B* **2021,** *9 (26),* 5264-5271.
2. 2. Bhatnagar, D.; Kumar, V.; Kumar, A.; Kaur, I., Graphene quantum dots FRET based sensor for early detection of heart attack in human. *Biosensors and Bioelectronics* **2016,** *79*, 495-499.
